# Methodological strategies for linking superordinate life goals (values) and daily activities: a cross-sectional online study of adolescents

**DOI:** 10.3389/fpsyg.2026.1685340

**Published:** 2026-03-17

**Authors:** Lawrence M. Scheier, William B. Hansen, Martin Komarc

**Affiliations:** 1 Prevention Strategies LLC, Greensboro, NC, United States; 2 LARS Research Institute, Sun City, AZ, United States; 3 Univerzita Karlova, Prague, Czechia

**Keywords:** activities, adolescence, life goals, psychometrics, values, weighting

## Abstract

Adolescents pursue life goals through daily activities that pave the way toward a future or ideal self. Very little is known, however, regarding what youth consider to be important activities in support of their life goals. In the present study, we examined four psychometric methods of weighting and rank ordering activities associated with six life goals (Fitness, Helpful, Smart, Talented, Creative, and Social). Cross-sectional data were obtained from an online survey of 2,001 youth ages 13 to 15 who picked a life goal and then rated whether 21 activities align with their life goal. Eleven of the activities for each goal were selected by ChatGPT and mixed with 10 filler activities (drawn from the remaining life goals). Four weighting methods including an ipsative distribution-based approach, confirmatory factor analysis, item response theory, and multidimensional scaling were used to rank order activities. The distribution method produced upper quartiles based on standard deviation width. Select activities were relatively concordant for six life goals using the four methods, underscoring the uniformity and convergence of these different approaches. All four methods provided evidence of the uniformity of the select 11 activities. Findings are discussed in terms of the composition of adolescent life goals, their rank-order consistency across different weighting schemes, and their conceptual importance as we learn more about what youth consider relevant activities as they pursue their life goals.

## 1 Introduction

It has long been suggested that values play an integral role in determining behavior including having motivational and goal-directed properties (Bardi and Schwartz, 2003; Rokeach, 1979). Values are considered instrumental in how people shape their life goals and idealized futures, acting as the “*engines*” that set into motion a person’s attitudes, beliefs, judgments, choices, attributions, and habitual behaviors (Rokeach, 1973). Schwartz (1994) considered values as enduring goals (i.e., desirable end states), while Rokeach (1973) suggested they are “modes of conduct” that can be both instrumental serving as standards to guide one’s day-to-day actions or terminal in terms of fostering enduring life pursuits. In the case of the former, one wishes to be “honest” or “helpful” as a way of living now and for the immediate future (i.e., instrumental values), whereas world peace is something worth striving for in the long-term (i.e., terminal values). When cast in terms of a person’s life goals, values are part of a person’s self-schemata and are strong contributors to the “*future self*” encompassing one’s wishes, hopes, and desires (Markus and Nurius, 1986). The pathways to achieving one’s values is obtained through pursuit of specific life goals (e.g., educational or occupational choices) that encompasses activities conducted daily (Massey et al., 2008). These life goals then become central to a person’s identity, constituting the actions they will take to achieve their desired life pursuits.

As important as life goals can be, not everyone meets their goals, and this can decidedly cause consternation. Rokeach (1973, 1979) specified that conflict with values, or discordant experiences, can occur when an individual confronts incongruity, inconsistencies, and contradictions between their own wants and desires (i.e., future goals) and either their own behavioral tendencies or societal standards. Resolving these incongruities, internalizing societal standards (i.e., norms), and minimizing discrepancies stimulates a person’s self-awareness and lays the foundation for developing consonance between their values and behaviors (Sagiv and Roccas, 2021). Values in this respect are part of a self-regulatory function (Carver and Scheier, 1982) and become central to a person’s social-cognitive interactions as well as being crucial to formulating their identity (Hitlin, 2003).

Despite the central role played by values in development, very little is known about how youth form their values, and what they consider as relevant activities in the pursuit of their life goals. For instance, if an adolescent considers being smart as an important life goal, what activities do they consider as relevant pursuits that support the goal of being smart. Some youth may feel that studying hard in school means being smart while others may think that playing chess or building robots means being smart. The same can be said about what youth consider important activities that support the goal of being talented. Here too, one youth may feel that painting on a canvass represents a unique talent, while another may suggest that skateboarding or jumping rope syncopated to music beats means being talented. Do the activities they choose for different life goals (e.g., being talented vs. being smart) overlap or are they distinct because the activities they may choose in support of life goals convey very different meanings? This would mean that what youth consider worthwhile pursuits to being smart is not the same as what they like doing in the pursuit of being talented. Conceivably, in their cognitive deliberation (i.e., internal representations) youth give different “weight” to one activity over another. These weights could determine how diligently youth will pursue a course of action to be smart or talented. Unfortunately, the idea of attaching empirical weights to different activities in light of a life goal has not been thoroughly addressed in the research literature.

In this study, we set out to learn more about the types of activities that adolescents associate with their life goals. This represents the conceptual side of our inquiry because there is not much in the literature about defining adolescent life goals. The second component of the study asks whether the activities that youth assign to different life goals can be weighted in some manner that supports a systematic approach. This involved contrasting standard psychometric approaches (e.g., factor analysis) with an ipsative distribution-based approach. We begin by briefly discussing the importance of values and their role in adolescent thinking. Then, we introduce four methods used to rank activities associated with one of six life goals that youth could choose. The latter is presented in a very nontechnical manner, but we refer readers to sources that can explain the underlying statistical proof. We then present empirical findings outlining the degree of convergence between the weighting methods and discuss what the different methods reveal about adolescent life goals and activities.

In the early years of development, during the period from late childhood to adolescence, values become the bedrock for a child’s interests and provide the substrate upon which they construct their lifestyle choices (Daniel et al., 2020). This can include forming friendships with individuals (i.e., peer selection) who share the same life goals or what is called consensual validation or homophily (Brechwald and Prinstein, 2011). In this instance, a child will want to play or engage in activities they can share with friends and that reinforce the “things they like to do” giving rise to the adage “birds of the same feather flock together” (McPherson et al., 2001). This can mean selecting friends that support athletic participation, including exercise or eating healthy (Benish-Weisman, 2024; Corte et al., 2022). It can also mean, in some instances, associating with deviant peers given the select activities that youth engage are either antisocial (Piehler and Dishion, 2007), risky (Jaccard et al., 2005), or run contrary to the accepted norm (Knecht et al., 2010). Values also reflect the strength of family bonds and socialization (Grusec and Davidov, 2007; Roest et al., 2010) and transmission of cultural mores from close influential people like teachers or clergy (Knafo and Schwartz, 2009). When these relationships are cast in a favorable (i.e., prosocial) light they can influence a developing child’s sense of morality and community (Sun and Shek, 2012). Values, in this example, become inextricably tied to what one does, what activities an individual pursues, and what they consider important to their future success and well-being.

Although there are several studies linking values with adolescent development (Daniel and Benish-Weisman, 2018; Daniel et al., 2020), very little is known about what youth value as life goals and what pursuits they believe foster these goals. The few studies of values in youth have either involved cross-sectional and cross-national studies (Casas et al., 2004; Ungvary et al., 2018), small sample qualitative studies that ask youth what they believe about a certain set of goals or preferred behaviors (Lewis-Smith et al., 2021; Strömmer et al., 2021) or studies of values in a specific context. The latter can include understanding values in the context of achievement in school framed by expectancy-value theory (Wigfield and Cambria, 2010; Wigfield and Eccles, 2000). Other studies conducted in a specific context have included school-based drug prevention to determine whether holding certain prosocial values diminishes susceptibility to drug use (Danioni et al., 2023; Hansen, 2021; Young and West, 2010). Collectively, these and related studies indicate that adolescence represents an ideal period to examine life goals. This is because during adolescence youth focus on crystallizing their identity (Erikson, 1968), they become more involved in educational pursuits, and they develop metacognitive skills that provide a basis for thinking about their life goals (Schneider, 2008). The maturation of cognitive and emotional skills that is part of formal operational reasoning enables them to engage in a form of perspective taking that provides a basis to consider what they wish to pursue in terms of their life goals.

As part of the second thrust of this study, we were interested in learning more about weighting schemes and ways to organize youth life goals along some continuum. This entailed contrasting four analytic methods (explained below) that each produce relative “weights.” At the heart of comparing the different weighting methods is an attempt to empirically address the issue of response sets. Response sets arise when individuals taking a test or survey acquiesce and choose favorable responses over unfavorable, fail to pay attention to the question, provide an arbitrary (inattentive or careless) response, or provide an “extreme” response in deference to some personality trait. Cronbach (1950) was one of the first to take note of response sets in testing situations. Since his seminal publication much has been written about how response sets can affect interpretation of personality assessments (Jackson and Messick, 1961) and other forms of both clinical and nonclinical assessment (McGrath et al., 2010). The focus in most of these publications is on social faking, desirability, and impression management, all examples of how people can overemphasize certain qualities to look good or appear consistent with the norm.

In the case of careless responding, participants are not paying attention to the content of the question, they have not read it fully or they lack motivation to fully digest the item content. Studies that specifically examine careless or inattentive responding point out that the greatest damage is to criterion-related or predictive validity with either inflation or attenuation of correlations (Meade and Craig, 2012). In the case of the former, this can increase the Type I error rate in hypothesis testing (reject the null when it is true), while in the case of the latter it can inflate the Type II error rate (accept the null when it is false). Because the underlying assumption in classical test theory is that a respondent’s test score consists of true variance and error (and the two terms are uncorrelated), in most cases and with high scale reliability, response sets that result from careless responding can be treated as nuisance variance (i.e., measurement error). With exceptionally large samples this should not be concerning. However, with smaller samples the presence of careless responding can be disruptive, bias standard errors, reduce reliability, attenuate correlations, and lead to spurious or confounding effects (Huang et al., 2015).

The ipsative distribution-based method that we test in the current study, which employs within-participant variability, is one means of addressing response set. As a means of weighting responses to each activity, it seeks to eliminate youth who were not taking the survey seriously (i.e., careless or inattentive responders) and distinguish them from those that read each question and gave it thought before answering (i.e., careful responders). This makes it possible to identify and eliminate youth who might repetitively choose the same number for each question without giving the question any further consideration (i.e., tapping the same key for all answers).

To derive the distribution-based method, we calculated the within-person across-item standard deviation (WSD) based on the ratings for all 21 items in each life goal (i.e., equivalent to a standard error of measurement)^1^. The underlying logic for this approach was that those who had larger WSDs were thought to be more discriminating in how they rated each activity. Activities that should be part of the life goal should receive a “4” or “5,” indicating agreement between the activity and the life goal. Conversely, filler activities that were not aligned with the life goal should receive a “1” or “2,” indicating disagreement. We reasoned that those individuals whose ratings had greater variability reflected greater attentiveness on their part to which activities fit which life goal. They were, in essence, paying closer attention, making an effort to think about each activity, and differentiating which activities belong to their chosen life goal. We took the top 25th percentile of cases based on their magnitude of their WSD for further analysis using this method. We then rank ordered participants’ mean ratings of the top 11 activities for each life goal.

This ipsative distribution-based method of ranking the top 11 activities was then compared to two additional “tried and true” regression-based methods of weighting. The first approach used confirmatory factor analysis (CFA) and the second approach used item response theory (IRT). In the case of the former approach, CFA is a type of common factor analysis derived from classical test theory that provides weights in the form of factor loadings (lambda or *λ*). These weights can be interpreted as indicating the magnitude or strength of the relationship between an unobserved latent construct (i.e., factor = life goal) and its associated items (i.e., activities). High loadings indicate the item in question makes a large contribution to the variance underlying the latent construct. The weights (factor loadings) in question are obtained by regressing the activity items on the latent construct producing slopes of regression lines as factor loadings (λ). One can then interpret the meaning or definition of the latent construct in terms of the contributions made by the full set of items conveyed by their weights.

IRT comes from modern test theory where the focus rests with an item’s “performance” and its ability to indicate a latent trait (theta or *θ*). This slightly different psychometric approach suggests a different model parameterization than CFA and puts item properties on the same logit scale as the underlying trait. The 2-PL IRT method used in the current study provides two pieces of relevant information including an item’s discrimination and difficulty parameters. The discrimination parameter (good fitting items range from ~1.0–3.0) reflects the slope of an items’ item characteristic curve (ICC), in other words, an item’s ability to discern “test” performance (where theta is an ability, understanding or performance). Difficulty (good fitting items range from ~-3.0 to 3.0), on the other hand, is the value of theta at which there is a 50% probability to answer the item correctly (indicating the underlying trait). Higher positive difficulty parameters indicate that the test taker required higher levels of the underlying ability (trait) to have a 50% probability of answering the question correctly^2^. Both CFA and IRT make assumptions, one of which is that the underlying latent construct is unidimensional, which we test in this study.

In addition to the two psychometric approaches already mentioned we also included multidimensional scaling (MDS) as an effective means of visualizing the “similarity” between select activities. MDS is also a form of data reduction that examines the Euclidean distance between items (i.e., a quantitative estimate of proximity) and that can be arranged on a spatial map depicting (dis)similarity. The resulting “empirical coordinate estimates” visually capture how closely aligned or “similar” activities are in relation to each other (Davison and Sireci, 2000). In the current context, the coordinates are used as weights indicating similarity (or dissimilarity) among the 21 Life Goal activities and can provide a means to reinforce when activities belong to some underlying dimensional space. This is akin to a principal component analysis^3^.

## 2 Materials and methods

### 2.1 Participants

Youth ages 13 to 15 enrolled in the TeenVoice’s EvolveMe® educational platform were invited to participate in this project between January and March of 2025. The TeenVoice’s EvolveMe® platform is operated as a subsidiary of the national non-profit *Britebound™* and provides opportunities for youth to acquire skills to boost their academic performance and acquire insight to their future aspirations including educational and vocational opportunities. In exchange for reward points participants can earn and use within the site, they agreed to complete a brief survey about their life’s goals and the activities that might support these goals. All youth were provided an e-assent on the TeenVoice’s EvolveMe® platform. Use of the platform is contingent on youth signing a “Terms and Conditions” e-document that stipulate rules and regulations and consent procedures. The author’s institutional review board (IRB 00011690), which is comprised of individuals not connected to the research and that have no financial interests in the grant funding or operations of the company, made a determination that the study was exempt and issued a waiver of documentation of consent. This decision comports with Health and Human Services (HHS) and US federal guidelines for protecting human subjects in research (45 CFR §46.104), the *Ethical Principles and Guidelines for the Protection of Human Subjects of Research* outlined in the Belmont Report, and US federal guidelines for a waiver of documentation of consent (CFR §46.116). The study did not involve procedures for which written consent is normally required outside of the research context^4^.

The TeenVoice’s EvolveMe® platform attracts a nationally representative distribution of teens across age, gender, race/ethnicity, and region of the country. Over the three-month recruitment period, two thousand one (2001) teens agreed to participate. The sample consisted of 1,019 females (50.9%), 948 males (47.4%) and 34 individuals (1.7%) who identified as non-binary. Slightly more than half (57.0%) identified as White youth, 19.3% identified as African American youth, 14.7% identified as Hispanic youth, 3.2% identified as Asian youth, and 5.7% identified as Other. Based on purposive sampling there were 564 (28.2%) 13-year-old youth, 781 (39.0%) 14-year-old youth, and 656 (32.8%) 15-year-old youth. Participants came from 51 states and territories with the most populous respondent states being California (154), Texas (154), Florida (150), Illinois (110), and Connecticut (72).

### 2.2 Instruments

The survey contained a single item that asked participants to select a life goal from among six options: Fitness, Helpful, Creative, Smart, Social, or Talented. These six options had been selected because of both earlier research (Hansen, 2021) and interviews with a different set of youth who reviewed and critiqued the initial list of life goals^5^.

For each of the six life goals, we generated a list of activities that might potentially be aligned with it. Potential activities were the product of ChatGPT-generated text (OpenAI, 2025). For example, “What can teens who want to develop a reputation for being *Helpful* do on a regular basis?” For *Talented*, we queried, “What can teens who want to be recognized for their talents and unique abilities routinely do to become talented?” and for *Smart*, we queried, “What can teens who have the goal of becoming smart and intelligent routinely do to enhance their thinking, memory, and insightfulness?” This type of query resulted in a total of 66 activities, 11 for each life goal. Once participants identified a life goal, subsequent items asked them to rate how important an activity was to accomplishing that goal. Participants rated activities on a 5-point Likert scale ranging from (1) “*Not helpful*” to (5) “*Very helpful*.” The list of activities presented to youth were randomized within each of the six life-goal-specific surveys. To create possible filler or distraction items, two items from each of the non-target life goals were selected to be included in the list presented to youth. Thus, in addition to the 11 aligned activities, an additional set of 10 non-aligned filler activities were also included in each life goal survey. Non-aligned items were selected in a quasi-random fashion.

### 2.3 Analysis

Data management and analyses for the distribution-based weighting scheme were conducted using IBM SPSS Statistics. The CFA analyses were conducted using the statistical package Mplus as were the IRT analyses (Muthén and Muthén, 2017). The MDS analyses were conducted using the SPSS PROXSCAL procedure.

## 3 Results

The online survey provided time stamps to record start and finish times (in seconds). Average time to complete the survey with the full sample (*n* = 2001) was $\bar{X}$ = 822.55 s (SD = 9762.83, min = 33, max = 277,356). Surveys with exceptionally long durations potentially reflect participants failing to submit their surveys when completed. Truncating the top end-to-completion cases to be 20 min or less made the average $\bar{X}$ = 143.967 s, about 2½ minutes (SD = 116.63, max = 1,204).

The selection of life goals indicated that 20.8% (416) chose Smart, 19.1% (383) chose Creative, 18.4% (369) chose Fitness, 14.4% (288) chose Helpful, 13.6% (273) chose Social, and 13.6% (272) chose Talented. A test for dependence between gender and life goal was not significant, χ^2^(6) = 11.41, *p* = 0.076, as was a test for dependence between age and life goal, χ^2^(12) = 11.97, *p* = 0.448. A test for dependence between race (white vs. nonwhite) and life goal was significant, χ^2^(6) = 14.95, *p* = 0.05, albeit samples were too small to test specific activities by race.

Table 1 show the reliability estimates using both McDonald’s omega (*ω*) statistic and Cronbach’s alpha for the 21 (11 targeted and 10 filler) activities for each life goal. In all cases, reliability estimates (both omega and alpha) were above the accepted benchmark of 0.70 (Nunnally and Bernstein, 1994).

**Table 1:** Estimates of reliability based on sample and item composition.

Items	Cases	Creativity	Fitness	Helpful	Smart	Social	Talented
All 21	All Cases	0.900 (0.902)	0.848 (0.796)	0.890 (0.889)	0.905 (0.906)	0.890 (0.891)	0.876 (0.873)
Targeted 11	All Cases	0.850 (0.855)	0.887 (0.891)	0.906 (0.906)	0.866 (0.866)	0.853 (0.855)	0.879 (0.881)
Top Ranked 11	All Cases	0.844 (0.851)	0.912 (0.888)	0.903 (0.904)	0.875 (0.876)	0.870 (0.871)	0.880 (0.882)
All 21	Upper Q (25%)	0.673	0.572	0.658	0.768	0.660	0.661
Targeted 11	Upper Q (25%)	0.642	0.694	0.887	0.740	0.716	0.858
Top 11	Upper Q (25%)	0.628	0.731	0.878	0.784	0.771	0.853

Numbers in parentheses are McDonald’s omega (*ω*).

Table 2 shows the results of CFA models for the full set of 21 activities and the reduced set of the top 11 ranked activities. All models posited simple structure with a single latent construct and were run using maximum likelihood estimation with robust standard errors (to address any nonnormality). Turning first to the fit indices with all 21 activities, model fit was less than adequate based on benchmark statistics (Hu and Bentler, 1999). This is expected, however, given that the full set included “filler” items that should not load on the life goal. Once filler items are removed, and the underlying factor is reflected by the primary activities, model fit improved considerably. Various fit indices like the Standardized Root Mean Residual (SMSR) and the Root Mean Square Error of Approximation (RMSEA) shrunk appreciably and the Comparative Fit Index (CFI) improved (higher values indicate better reproduction of the covariance matrix relative to a baseline model specifying null relations. The far-right column shows the average factor loadings (lambdas or *λ*) for the 21 activities for each life goal and the corresponding 11 highly ranked activities for the same constructs. The results reinforce that, for the most part, removing filler activities improved the psychometric properties of each CFA model.

**Table 2:** Fit indices for full set of 21 activities and reduced set of top 11 ranked items.

Model	CFI	χ^2^/df	RMSEA (90% CI)	SRMR	λ_avg._^1^
Creative 21(383)	0.797	717.081/189	0.085 (0.079 0.092)	0.062	0.548
Creative 11	0.901	154.385/44	0.081 (0.067 0.095)	0.047	0.574
Fitness 21 (369)	0.636	1374.609/189	0.130 (0.124 0.137)	0.149	0.428
Fitness 11	0.960	123.798/44	0.070 (0.056 0.085)	0.033	0.698
Talented 21 (272)	0.733	660.831/189	0.096 (0.088 0.104)	0.093	0.489
Talented 11	0.941	106.875/44	0.072 (0.055 0.090)	0.040	0.631
Social 21 (273)	0.827	474.282/189	0.074 (0.066 0.083)	0.064	0.527
Social 11	0.949	91.533/44	0.063 (0.045 0.081)	0.039	0.614
Helpful 21 (288)	0.694	900.355/189	0.114 (0.107 0.122)	0.115	0.505
Helpful 11	0.972	81.708/44	0.055 (0.036 0.073)	0.032	0.678
Smart 21 (416)	0.850	627.151/189	0.075 (0.068 0.081)	0.054	0.564
Smart 11	0.926	156.096/44	0.078 (0.065 0.092)	0.042	0.622

^1^Average standardized loading (lambda) across full set of items.

Table 3 shows the results of comparing the four weighting methods (ipsative distribution-based sample weights, CFA, IRT, and MDS) for the life goal Fitness. The distribution-based percentile weighting approach for the ranking of the top 11 activities is based on a reduced sample (upper quartile) whereas the CFA, IRT, and MDS approaches use the full sample of youth who chose this life goal. This avoids estimation problems that would arise with a small sample where the number of parameters in the model might exceed the number of cases. Fit of the CFA model was determined using cut-off criteria for inferential fit indices including the CFI, SMSR, and RMSEA (Hu and Bentler, 1999). Although these statistics are important to evaluate the fit of a hypothesized model against the sample data, obtaining a well-fitting model is not the sole focus of this study (e.g., improving model fit by adding correlated residuals through specification searches, which can be unstable with small samples; MacCallum, 1986). In the current context, the focus is on the magnitude of factor loadings and how well they compare across the different weighting methods. For the MDS solution, goodness of fit was based on the Kruskal (1964) measure of stress (smaller values are better indicating less distance between stimulus pairs) and Tucker’s Coefficient of Congruence (CoC; Lorenzo-Seva and ten Berge, 2006) where larger values are better. Stress is a normalized, least squares index of mismatch between the estimated distances and the transformed data (input proximities). It is based on a maximum likelihood optimization function with a designated number of iterations. The Tucker CoC is the cosine of the angle between two vectors and is a standardized measure of how well two factor loading matrices (raw and transformed) converge using a Procrustes rotation. Higher values indicate the factor loadings are converging, and the two dimensions can be considered equivalent.

**Table 3:** Activity rankings by the four methods for the fitness life goal.

Variable	Activity	FIT	Mean	SD	Top 21	Top 11	Lambda	IRT-DS	IRT-DF	MDS
Fit_7	Eat healthy food and have a balanced diet	Primary	4.03	1.13	21	**11**	**0.715**	2.322	−0.713	−0.651
Fit_9	Get enough sleep	Primary	4.03	1.12	20	**10**	**0.717**	2.006	−0.648	−0.657
Fit_17	Run, jog, ride a bicycle, or swim	Primary	3.99	1.16	19	**9**	**0.726**	2.655	−0.591	−0.623
Fit_15	Practice daily	Filler	3.98	1.14	18	**8**	**0.759**	2.486	−0.602	−0.662
Fit_6	Drink enough liquids	Primary	3.91	1.24	17	**7**	**0.715**	1.929	−0.548	−0.613
Fit_10	Hike or walk	Primary	3.88	1.13	16	**6**	**0.658**	1.967	−0.553	−0.534
Fit_4	Do push-ups, squats, lunges, and planks	Primary	3.85	1.29	15	**5**	**0.790**	2.505	−0.437	−0.644
Fit_14	Play on a sports team	Primary	3.79	1.16	14	**4**	**0.656**	1.637	−0.464	−0.537
Fit_11	Lift weights	Primary	3.77	1.26	13	**3**	**0.742**	2.202	−0.32	−0.617
Fit_20	Stretch or do yoga	Primary	3.59	1.23	12	**2**	**0.523**	1.32	−0.26	−0.345
Fit_3	Do exercises like jumping jacks	Primary	3.57	1.23	11	**1**	**0.608**	1.508	−0.139	−0.458
Fit_1	Borrow equipment to support your talent	Filler	3.27	1.23	10		**0.567**	1.518	0.238	−0.235
Fit_8	Explore mutual interests you have with others	Filler	3.22	1.21	9		0.142	0.545	0.524	0.355
Fit_18	Share tools and equipment you own	Filler	3.00	1.30	8		0.061	0.444	1.267	0.550
Fit_2	Compose music	Filler	2.88	1.35	7		0.056	0.582	1.126	0.560
Fit_21	Volunteer at a local charity	Filler	2.87	1.37	6		−0.042	0.387	1.502	0.764
Fit_12	Participate in dance or cheerleading	Primary	2.78	1.29	5		0.211	0.952	1.016	0.482
Fit_16	Read books about science and philosophy	Filler	2.61	1.28	4		−0.116	0.319	3.263	0.901
Fit_13	Perform your talent in front of others whenever you can	Filler	2.57	1.34	3		−0.029	0.366	2.864	0.901
Fit_19	Solve puzzles and riddles	Filler	2.57	1.33	2		−0.117	0.37	2.872	0.936
Fit_5	Draw sketches of people or things	Filler	2.51	1.40	1		−0.193	0.051	17.5	1.127

*N* = 369. SD, standard deviation; IRT-DS, item response theory discrimination parameter; IRT-DF, item response theory difficulty parameter; Lamdba, standardized factor loading from CFA model positing simple structure; MDS, multidimensional scaling location parameter. Bold values indicate top ranked 11 activities selected by youth that comport with the life goal.

As Table 3 (*Fitness*) shows, for most of the items there was very good correspondence between the different weighting methods. The factor loadings from the CFA indicated that only one filler item (practice daily) was ranked 10th by the quartile standard deviation method and had a high factor loading (*λ* = 0.765). One item (participating in dance or cheerleading) was a primary activity proposed by ChatGPT and ranked 8th in the full list of 21 activities with a factor loading below the acceptable threshold (λ = 0.211). Items that fail to achieve congruence between the methods are most likely either worded in a nebulous fashion and can fit more than one activity (e.g., practicing is also associated with the life goal *Talented*), or because youth in this age group do not associate the activity strongly with their fitness goals.

The IRT method also provides several pieces of information about the 21 activities and their correspondence to the latent trait of *Fitness*. First, there is a lot of consistency in the various IRT parameters that indicate the strength of an activity in discerning *Fitness*. For instance, there is a marked decrease in the sheer magnitude of the discrimination (slope) parameter after the top 11 ranked activity items. Higher discrimination parameters suggest that the item performs well to discriminate individuals in a narrow high-end band of theta (latent trait), in other words, the youth have a clear picture of what activities are part of *Fitness*. With few exceptions, discrimination parameters for the filler items indicate that many items do not discern the difference between respondents who had a grasp of which activities support *Fitness* and those that do not. The difficulty (location) parameter was relatively small and negative for the top 11 activities. This means that even at relatively low levels of the trait (average levels of theta or knowledge of *Fitness*), on average respondents had a greater than 50% probability of being able to pick these 11 activities as associated with *Fitness*. Importantly, the transition from negative to positive valence and the relatively larger difficulty parameters (meaning higher levels of the trait of knowing what defines *Fitness* was required) occurred after the top 11 ranked items. In only one case was there a conflict between the intended activity and its ranking. This was for “practice daily,” which had a high discrimination parameter but was a filler item (2.486). Respondents may have associated “top of mind” their goal of *Fitness* requires they “practice a sport” daily in order to excel. Taken together, these parameters indicate that the primary (ChatGPT proposed) activities yielded substantial information about *Fitness* and that youth were able to select relevant activities associated with their *Fitness* life goal.

**Figure 1:**
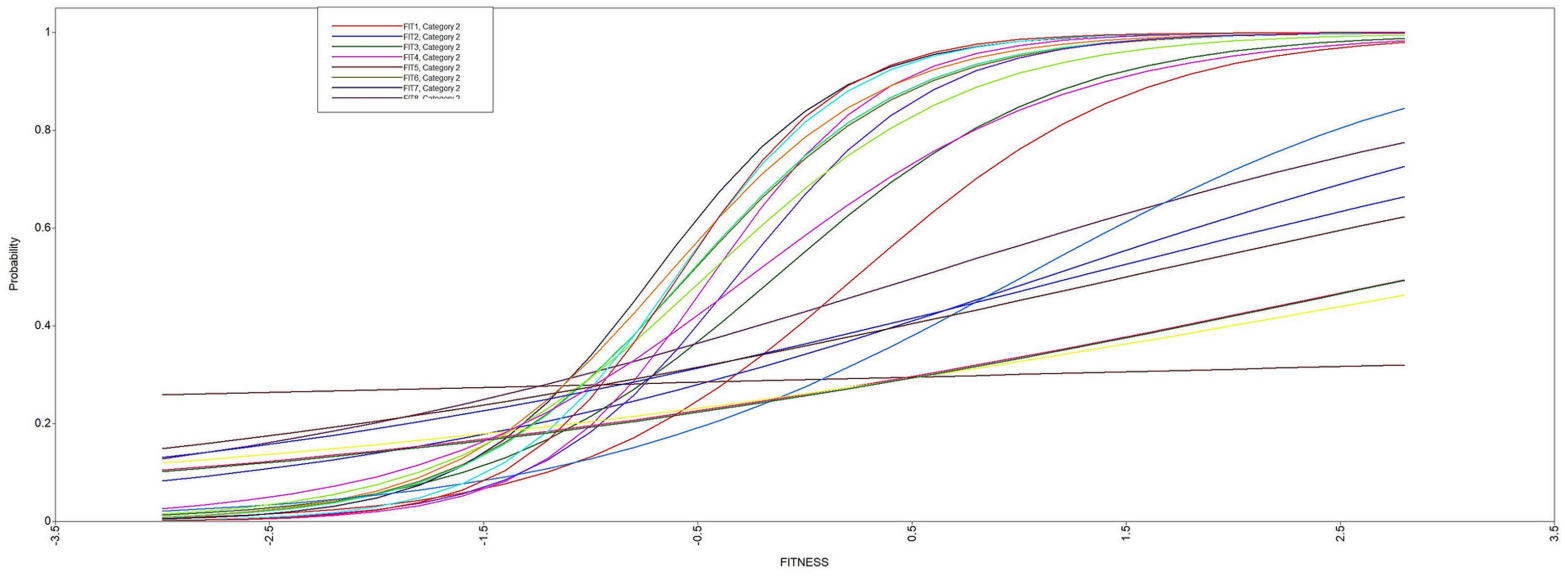
Item characteristic curve (ICC) for the fitness life goal 21 activities.

Figure 1 shows the item characteristic curve (ICC) for the Fitness life goal for all 21 items. The ogive shape of the curve characterizes the relationship between the underlying trait (knowing which items are associated with *Fitness*) and each of the individual activities. In this case, the probability of choosing the correct activities increased with higher levels of the latent trait. The slight shift of items to the left of the midpoint (−0.5) shows that items slightly below the mean level of theta also did a good job of discriminating whether the respondents had a clear picture of which activities constitute *Fitness*.

**Figure 2:**
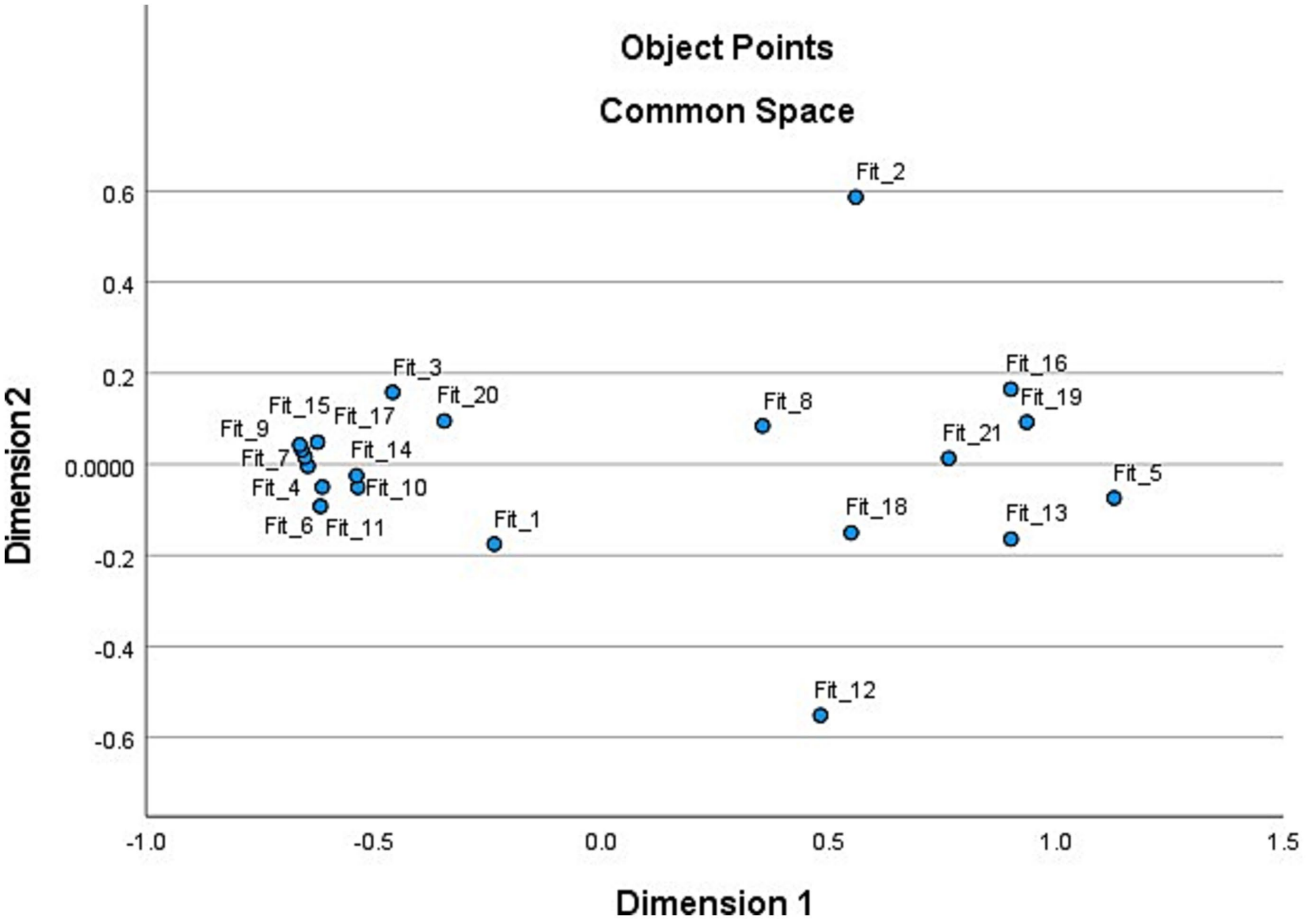
MDS coordinates for the 21 fitness life goal activities.

The MDS analysis indicated a good fit, with a relatively low S-Stress value (0.00173) and a high Tucker’s CoC (0.99878). Table 3 also shows the MDS coordinate estimates for the 21 items (and the reduced set of 11). The coordinate estimates for the top 11 ranked items for the full set of 21 activities were all located in the same quadrant of the plot (all negative with similar proximity locations). Figure 2 shows the coordinates for the full set of 21 activities plotted in a 2-dimensional space. As depicted, there was a considerable bunching of the fitness activities in the left quadrant including the 11 primary activities that belonged to the *Fitness* life goal.

Supplementary Table 1 shows the same results for the *Helpful* life goal. The average CFA loading for all 21 activities was *λ* = 0.505 whereas this number increased to λ = 0.678 when only the top ranked 11 activities were modeled (i.e., a more homogenous set of activities indicating the underlying factor). The IRT analysis also shows that the top 11 ranked activity items had high levels of discrimination and the small negative difficulty parameters indicate the activities selected by youth worked well to discriminate the trait (*Helpful*) and youth found it relatively easy to pick activities that indicate this life goal. Supplementary Figure 1 shows the ogive-shaped ICC for the 21 Helpful activities. Like the *Fitness* ICC, the curve demonstrates that generally speaking, respondents with higher levels of the trait (knowledge of activities aligned with being *Helpful*) were likely to endorse the corresponding activities.

The MDS analysis also reinforced a good fit, with a relatively low S-Stress value (0.01265) indicating that activities that should hang together did so and had a high Tucker’s CoC (0.99484). Supplementary Table 1 shows the MDS coordinate estimates for the 21 items. Most of the coordinate estimates for the top 11 ranked items were in the same quadrant of the plot (negative with similar proximity locations). The exception was for activity #5 (give a lot of compliments), which was a “filler” item and had a small negative coordinate (−0.185). This indicates the item was not in the section of the map overlapping with the other activity items located in this quadrant. The one primary item that should have been proximal to the other activities was “be cheerful when someone asks for help,” which was outside the top 11 ranked items but based on the statistical information from the three methods (CFA, IRT, MDS), should have been ranked. Supplementary Figure 2 shows the MDS coordinates for the 21 Helpful life goal activities plotted in a 2-dimensional space. All 11 of the primary (ranked) activities are in proximity on the left-hand side of the plot.

Supplementary Tables 2–5 show the same activity weighting and ranking information for the *Creative, Smart, Social,* and *Talented* life goals (not discussed in the interest of conserving space). One important point is the degree of overlap between what was earmarked as “Primary” and should be considered as an activity associated with a life goal, and what was “Filler” and selected from different life goals to collectively test the veracity of the ranking (weighting) system. The overall hit rate for all six life goals was 73% where youth picked the activity that corresponded with the life goal. The highest success for hit rates was observed for *Fitness, Helpful*, and *Talented* (91% for all three, respectively) and this was followed in decreasing order by *Creative* (64%), *Smart* (55%), and *Social* (45%). The smaller hit rates indicate youth had difficulty aligning the activities with these life goals.

## 4 Discussion

The study of adolescent values is a very promising area of study given the importance of values in determining an individual’s pursuits and life goals. Values encompass the beliefs individuals hold that in both the short- and long-term will help them achieve their goals in life. Values are often considered stable “motivational” constructs, norm driven, socially desirable, and prompting individuals to act in certain ways, seek a particular course of action, and live their life by certain principles. Values are important at every stage of life from childhood forward and become especially significant in adolescence. This is because adolescents advance quite rapidly in their cognitive skills and begin to contemplate the future, thinking about what “will happen” in life and its many possible outcomes. Values become central to a youth’s identity and occupy a central role in their sense of selfhood helping them reconcile “who am I?” and “who will I become?” Values in this respect, act as guides to help youth prioritize their decision making and give youth a sense of purpose in a complex and rapidly changing world (Boldero and Francis, 2002).

Most studies of values provide a list of values and ask individuals to rank order them in terms of their personal significance (i.e., value expressive behaviors). Much of this effort stems from the seminal work of Schwartz and colleagues ascertaining what comprised core human values (Schwartz, 2012; Schwartz, 1994; Schwartz and Bilsky, 1987). In their original work, Schwartz and colleagues asked individuals to rate how much a particular value was ‘opposed to their principles’ or ‘of supreme importance.’ Their answers were then fit to a circumplex model based on congruency between value and value expressive behavior ultimately boiling down to 10 core values (e.g., power, security, conformity, benevolence, self-direction). Importantly for our own work, Schwartz used CFA and MDS to align the values into the circumplex model^6^.

We took a slightly different tactic and presented youth with six values that were culled from an online qualitative study of life goals. We then asked these youth to select one optimal life goal (most reflective of their own aspirations) and rate a list of 21 activities they felt would be congruent with their chosen goal (i.e., providing a measure of value-behavior congruency in the Schwartz tradition). Eleven of the activities supported pursuing the goal and 10 items were “filler” items that were randomly selected from the other five life goals. By so doing, we were hoping that youth could align specific activities with a life goal. If it is any indication of what youth in the 13 to 15 age category ‘value’ they chose *Smart* as the most popular goal and *Talented* as the least popular goal. Even though *Talented* was not ranked highly among the six goals, youth correctly a core set of activities that accompany being talented. The same was observed for *Creative* and *Helpful*, both ranked highly in the three life goals selected by the youth and for which they indicated a very clear subset of activities that would support this goal.

In addition to learning more about life goals in this age group, we also wanted to find out if four different weighting approaches would converge and provide statistical support for the activity ratings youth provided for their select life goal. In other words, we wanted to know whether youth picked certain activities that seemed closely aligned to their life goal or they did not have a clear picture or engaged in careless responding. Although there exist numerous ways to identify careless responding (Curran, 2016; Huang et al., 2012; Huang et al., 2015; Meade and Craig, 2012), we offered four techniques that provide a different psychometric angle on this concern. The ipsative distribution-based approach captured dispersion in response sets by using the upper quartile of ratings within-individual based on their standard deviation score for all 21 activities. This approach suggests that youth who read the question in earnest, who are attentive and gave it some thought, would be judicious in their rating, thinking carefully about which activities were or were not tied to their life goal. Those activities located in the upper quartile have the most spread, reflecting the youths’ willingness to rate the 21 activities in a fair and even-handed manner. From a survey methodology point of view, this represents one way to eliminate careless responding particularly among respondents who click the same keyed number for every answer. This is known as the ‘longstring’ or invariability phenomenon, which entails repeatedly hitting the same response option regardless of the item content and with the result of reducing dispersion (Meade and Craig, 2012).

The ipsative distribution-based method was compared to three tried and true psychometric methods that have a long history in the field of survey construction. Each of the psychometric methods assumes an underlying latent construct or trait that is indicated by imperfect measures. The CFA method produces factor loadings that indicate the strength of an activity as an indicator of a life goal, the latter posited as a latent construct. Careless responding would diminish the purity of a latent construct, owing to increased amounts of measurement error. The IRT method provides information on the utility and performance of an activity as an indicator of a latent trait, which in the current context is equivalent to knowing correctly which activities comprise a life goal. Here too, careless responding would diminish the performance of an item and render it less useful as an indicator of the respondent’s true latent trait. The MDS approach provides a geometric representation of the proximal closeness or “similarity” of activities. This information can then be used to visually inspect whether the relations between activities is sufficient to infer an underlying dimension. The goal here was to ascertain the degree of convergence between the different methods in pointing to a common set of activities that reflected each life goal.

Although we only explored two of the six life goals in depth, there was substantial empirical evidence that the four methods converged and reinforced each other. There was 73% accuracy in aligning activities with the six life goals, which is better than chance. With one or two exceptions, statistical parameters from each method all indicated that the top ranked activities were indicative of an underlying construct or trait and supported the desired congruence between activities and life goals. Loadings from the CFA were all high in magnitude for the top ranked 11 activities and the average of these loadings was higher for the 11 focal items when compared to the full set of 21 activities that included filler activities. The same for the IRT analyses, which underscored consistently high, positive discrimination parameters and relatively small and negative difficulty parameters for the top 11 ranked items compared to filler items. Taken as a whole, the different model parameters show the utility of the select activities as indicators of the latent trait. The MDS results also graphically showed the top 11 ranked activities generally fall into a common space occupying the same position in a quadrant and reflect an underlying common dimension. Overall, this multi-pronged approach gives us a much richer impression of what youth consider as vital activities to achieve their life goals.

It is worth noting that the methodology reported here was not without some mismatches. Mismatches occur when a primary activity that was selected to be concordant with a life goal was not ranked in the top 11. When this occurred, we examined the activity to see why it may not have been rated highly by the youth. In almost all cases, the item was written in a way that it could have applied to multiple goals or lacked a certain specificity. In the case of *Fitness*, the one activity (practice daily), which was ranked in the 8th position, could also fit *Talented* as a life goal. The item “participate in dance or cheerleading” was ranked 5th (lower is worse) but was a primary activity chosen by ChatGPT to reflect *Fitness*. It may very well be that this activity was appealing only to a segment of the sample who were female and who would be more inclined to think of cheerleading as a fitness activity^7^. The same held true for *Helpful*, which had one mismatch where a primary activity (be cheerful when someone asks for help) was not ranked in the top 11 (ranked 9th overall out of 21). This activity could also fall under the *Social* life goal.

### 4.1 Limitations

There are several limitations associated with this study that may limit generalizability. The survey sampled youth from one educational platform. Although usage patterns of the TeenVoice’s EvolveMe® educational platform mimic the US census data for youth (in terms of both race and gender), it still speaks only to those youth using the platform who are motivated to improve their futures. The survey allowed young people to only pick one life goal and thus the full sample of 2001 was delimited based on which of the six life goals was selected. This may have hindered the generalizability of the IRT models, which require large samples for stable parameter estimation. Testing IRT required that we dichotomize the activities at the midpoint to test a 2-PL model. This effort loses some pertinent information, but it is much easier to identify viable solutions and interpret the obtained parameters compared to an IRT model that has items with 5-point ordinal scaling.

The life goals we picked were suggested by focus group participants in the same age group but there are numerous other life goal related values, some that may be more familiar we could have sampled. We encouraged youth in the focus group interviews to discuss what they felt were relevant life goals.

Other limitations include the different statistical techniques we applied, which are a subset of a wide variety of psychometric and statistical approaches we could have used. Factor analysis results are extremely sensitive to careless responding, and this may have affected the solutions and corresponding weights. We did not make any comparisons based on age, gender, or race given the small samples that resulted from using the upper quartile method. This may affect the generalizability of these findings. Values and life goals that support those values may change over time and we did not have the luxury of longitudinal data to test change over time (Daniel and Benish-Weisman, 2018).

Even with these limitations the current study provides a novel conceptual and methodological perspective from which to view values for youth. Future studies can certainly find ways to increase the sample size and offer new schemes from which to weigh activities that youth find supportive of their life goals.

## 5 Conclusion

The methodology in the current study suggests several avenues of pursuit worth noting. First, the ipsative distribution-based approach provides one means of identifying careful versus careless responding using SD bandwidths. This strategy to identify response sets was reinforced by the other psychometric methods. The convergence among methods provides additional tools to identify items that may not suitably fit a designated life goal. We also gained insight into what youth believe are activities that fit certain life goals and the lack of complete independence of both activities and life goals. Some activities fit more than one life goal, perhaps pointing to a shared space where youth believe that certain activities will make them both creative and smart or talented and creative. The ability to rank order activities and determine their correspondence to a life goal provides insight to what youth believe is worth pursuing at this point in their life and can have ramifications for psychoeducational interventions that support life skills training.

## Data Availability

The raw data supporting the conclusions of this article will be made available by the authors, without undue reservation.

## Footnotes

1 This is the residual mean square in a one-way ANOVA and reflects variability within the individual or variability in the measurement process or both sources.

2 There are drawbacks to CFA in the way it handles item performance that can be addressed with IRT.

3 The rating scales used in the survey are ordinal making the MDS analysis non-metric. The Euclidean distance functions (coordinate estimates) are disparities or rank images of the data, reinforcing their numerical assignment can be used as weights to capture rank order in terms of their proximity to the underlying dimension.

4 Specifically, the study is survey based, not an intervention, poses no more than minimal risk to participants, and did not involve any assessment of high-risk or illegal behaviors. No identifying information was collected by the research team, and all data was stripped of any personal identifiers on the EvolveMe® platform (prior to conveying the data electronically to the researchers). The nature of the data collected is limited to participants’ subjective evaluation of their life goals and activities associated with these goals--information that does not implicate sensitive or private matters.

5 Participants in all phases of this research were part of an NIAAA-funded SBIR Phase I grant (1R43AA031941–01) to create an online alcohol prevention program based on values clarification.

6 Even with the child version of the original adult survey of values, Schwartz queried of respondents “how much like you is this person?” The similarity between oneself and the portrait goals and aspirations is used as a gauge of values consistency.

7 The samples were too small to robustly test gender differences in the CFA loadings using a multigroup model specifying factorial invariance (and given the number of parameters with 21 activities).
