# Supplementary material for: Methodological strategies for linking superordinate life goals (values) and daily activities: a cross-sectional online study of adolescents
Source: Front Psychol. 2026 Mar 17;17:1685340. doi: 10.3389/fpsyg.2026.1685340 (PMC13036117; doi:10.3389/fpsyg.2026.1685340)
Supplement: Supplementary file 1 [file Data_Sheet_1.zip › Supplemental Table 3 Smart Activities.docx]

| **Supplemental Table 3.**  *Activity Rankings by the Four Methods for the Smart Life Goal* | | | | | | | | | | |
| --- | --- | --- | --- | --- | --- | --- | --- | --- | --- | --- |
|  |  |  |  |  |  |  |  |  |  |  |
| **Variable** | **Activity** | **FIT** | **Mean** | **SD** | **Top 21** | **Top 11** | **Lambda** | **IRT-DS** | **IRT-DF** | **MDS** |
| Smart_9 | Learn a new language | Primary | 3.71 | 1.20 | 21 | **11** | **0.630** | 1.000 | -0.407 | -0.267 |
| Smart_4 | Get enough sleep | Filler | 3.69 | 1.23 | 20 | **10** | **0.560** | 1.403 | -0.365 | -0.122 |
| Smart_3 | Gather and analyze data | Filler | 3.66 | 1.27 | 19 | **9** | **0.613** | 1.617 | -0.305 | -0.385 |
| Smart_5 | Give advice when someone asks for it | Primary | 3.64 | 1.16 | 18 | **8** | **0.527** | 1.196 | -0.312 | 0.000 |
| Smart_17 | Take lessons from a talented teacher | Primary | 3.62 | 1.24 | 17 | **7** | **0.653** | 1.777 | -0.243 | -0.232 |
| Smart_16 | Solve puzzles and riddles | Filler | 3.60 | 1.21 | 16 | **6** | **0.597** | 1.273 | -0.332 | -0.548 |
| Smart_10 | Memorize lists of numbers and facts | Filler | 3.58 | 1.19 | 15 | **5** | **0.582** | 1.393 | -0.124 | -0.14 |
| Smart_15 | Read books or articles about inventions | Filler | 3.57 | 1.23 | 14 | **4** | **0.672** | 1.862 | -0.239 | -0.383 |
| Smart_20 | Write down your thoughts and ideas | Primary | 3.56 | 1.18 | 13 | **3** | **0.601** | 1.393 | -0.229 | -0.358 |
| Smart_18 | Talk a lot to other smart people | Primary | 3.55 | 1.27 | 12 | **2** | **0.617** | 1.454 | -0.206 | -0.488 |
| Smart_6 | Help someone with their homework | Primary | 3.50 | 1.20 | 11 | **1** | **0.609** | 1.475 | -0.214 | -0.105 |
| Smart_19 | Watch education programs and documentaries | Filler | 3.49 | 1.24 | 10 |  | **0.666** | 1.827 | -0.173 | -0.458 |
| Smart_14 | Read books about science and philosophy | Primary | 3.46 | 1.27 | 9 |  | **0.723** | 2.239 | -0.039 | -0.271 |
| Smart_12 | Play games like chess and strategy games | Filler | 3.43 | 1.26 | 8 |  | **0.585** | 1.251 | 0.013 | -0.017 |
| Smart_8 | Join a club, team, or music ensemble | Primary | 3.38 | 1.21 | 7 |  | 0.443 | 0.955 | 0.019 | 0.628 |
| Smart_1 | Ask a lot of questions | Filler | 3.36 | 1.16 | 6 |  | **0.536** | 1.271 | 0.185 | 0.275 |
| Smart_21 | Write stories or poems | Filler | 3.29 | 1.20 | 5 |  | 0.401 | 0.857 | 0.246 | -0.293 |
| Smart_13 | Read about creative people | Filler | 3.25 | 1.19 | 4 |  | **0.592** | 1.282 | 0.379 | -0.258 |
| Smart_7 | Introduce yourself to people you do not know | Primary | 3.24 | 1.19 | 3 |  | 0.219 | 0.405 | 0.900 | 1.342 |
| Smart_11 | Play challenging computer games | Primary | 3.14 | 1.19 | 2 |  | **0.542** | 1.346 | 0.471 | 0.321 |
| Smart_2 | Drink enough liquids | Primary | 3.12 | 1.29 | 1 |  | 0.326 | 0.83 | 0.527 | 1.548 |
| *Note*: N = 416. SD = standard deviation; IRT-DS = item response theory discrimination parameter; IRT-DF = Item response theory difficulty parameter; Lambda = standardized factor loading from CFA model positing simple structure; MDS = multidimensional scaling location parameter. Bold numbers indicate top ranked activities. | | | | | | | | | | |
