# Supplementary material for: Methodological strategies for linking superordinate life goals (values) and daily activities: a cross-sectional online study of adolescents
Source: Front Psychol. 2026 Mar 17;17:1685340. doi: 10.3389/fpsyg.2026.1685340 (PMC13036117; doi:10.3389/fpsyg.2026.1685340)
Supplement: Supplementary file 1 [file Data_Sheet_1.zip › Supplemental Table 4 Social Activities.docx]

| **Supplemental Table 4.**  *Activity Rankings by the Four Methods for the Social Life Goal* | | | | | | | | | | |
| --- | --- | --- | --- | --- | --- | --- | --- | --- | --- | --- |
|  | | | | | | | | | | |
| **Variable** | **Activity** | **FIT** | **Mean** | **SD** | **Top 21** | **Top 11** | **Lambda** | **IRT-DS** | **IRT-DF** | **MDS** |
| Social_16 | Listen to others when they talk | Primary | 3.71 | 1.27 | 21 | **11** | **0.677** | 1.000 | 0.391 | -0.710 |
| Social_17 | Remember birthdays and other special days | Filler | 3.63 | 1.22 | 20 | **10** | **0.570** | 1.416 | 0.095 | -0.785 |
| Social_6 | Explore mutual interests you have with others | Primary | 3.61 | 1.15 | 19 | **9** | **0.627** | 1.627 | -0.123 | -0.417 |
| Social_8 | Give a lot of compliments | Primary | 3.56 | 1.18 | 18 | **8** | **0.542** | 1.606 | -0.200 | -0.273 |
| Social_4 | Be cheerful when someone asks for help | Primary | 3.54 | 1.14 | 17 | **7** | **0.622** | 2.286 | 0.727 | -0.300 |
| Social_3 | Be a good listener when someone needs help | Filler | 3.53 | 1.25 | 16 | **6** | **0.677** | 1.672 | -0.232 | -0.585 |
| Social_21 | Volunteer to help with special events | Filler | 3.53 | 1.25 | 15 | **5** | **0.554** | 1.529 | 0.066 | -0.246 |
| Social_14 | Join a club, team, or music ensemble | Primary | 3.49 | 1.23 | 14 | **4** | **0.637** | 1.809 | -0.207 | -0.394 |
| Social_13 | Introduce yourself to people you do not know | Filler | 3.38 | 1.23 | 13 | **3** | **0.626** | 1.538 | 0.188 | -0.456 |
| Social_7 | Find opportunities to share your talents | Filler | 3.37 | 1.20 | 12 | **2** | **0.528** | 0.958 | 0.464 | -0.145 |
| Social_2 | Attend social events and parties | Filler | 3.34 | 1.25 | 11 | **1** | **0.540** | 1.067 | 0.062 | -0.632 |
| Social_9 | Give small gifts | Primary | 3.32 | 1.17 | 10 |  | **0.533** | 1.163 | 0.453 | 0.141 |
| Social_11 | Help with raising money for good causes | Filler | 3.32 | 1.26 | 9 |  | **0.609** | 1.688 | 0.064 | -0.096 |
| Social_1 | Ask others about their hobbies and interests | Filler | 3.25 | 1.19 | 8 |  | **0.579** | 1.130 | -0.029 | -0.158 |
| Social_12 | Hike or walk | Primary | 3.22 | 1.17 | 7 |  | 0.385 | 0.912 | 0.705 | 0.206 |
| Social_19 | Take photos of people or things | Filler | 3.19 | 1.22 | 6 |  | 0.377 | 0.860 | -0.515 | 0.548 |
| Social_20 | Teach others about your talent | Primary | 3.19 | 1.24 | 5 |  | 0.462 | 1.078 | -0.265 | 0.518 |
| Social_10 | Help neighbors with cleaning their home | Primary | 3.16 | 1.25 | 4 |  | 0.342 | 0.736 | 0.711 | 0.78 |
| Social_5 | Eat healthy food and have a balanced diet | Primary | 3.12 | 1.24 | 3 |  | 0.377 | 0.669 | 0.452 | 0.482 |
| Social_15 | Learn to play a musical instrument | Primary | 2.97 | 1.36 | 2 |  | 0.381 | 1.042 | 0.386 | 1.366 |
| Social_18 | Run for a school or class office | Filler | 2.95 | 1.29 | 1 |  | 0.414 | 1.068 | -0.143 | 1.155 |
| *Note*: N = 273. SD = standard deviation; IRT-DS = item response theory discrimination parameter; IRT-DF = Item response theory difficulty parameter; Lambda = standardized factor loading from CFA model positing simple structure; MDS = multidimensional scaling location parameter. Bold numbers indicate top ranked activities. | | | | | | | | | | |
