# Supplementary material for: Methodological strategies for linking superordinate life goals (values) and daily activities: a cross-sectional online study of adolescents
Source: Front Psychol. 2026 Mar 17;17:1685340. doi: 10.3389/fpsyg.2026.1685340 (PMC13036117; doi:10.3389/fpsyg.2026.1685340)
Supplement: Supplementary file 1 [file Data_Sheet_1.zip › Supplemental Table 5 Talented Activities.docx]

| **Supplemental Table 5.**  *Activity Rankings by the Four Methods for the Talented Life Goal* | | | | | | | | | | | |
| --- | --- | --- | --- | --- | --- | --- | --- | --- | --- | --- | --- |
|  | | | | | | | | | | | |
| **Variable** | **Activity** | | **FIT** | **Mean** | **SD** | **Top 21** | **Top 11** | **Lambda** | **IRT-DS** | **IRT-DF** | **MDS** |
| Talented_19 | | Set goals for improving your talent | Primary | 3.67 | 1.20 | 21 | **11** | **0.738** | 1.000 | 0.672 | -0.573 |
| Talented_20 | | Take lessons from a talented teacher | Primary | 3.64 | 1.17 | 20 | **10** | **0.692** | 1.679 | 0.506 | -0.546 |
| Talented_13 | | Practice the talent you wish to have daily | Primary | 3.53 | 1.23 | 19 | **9** | **0.673** | 1.676 | 0.131 | -0.505 |
| Talented_18 | | Seek feedback when you perform for others | Primary | 3.46 | 1.21 | 18 | **8** | **0.626** | 1.933 | 0.016 | -0.339 |
| Talented_4 | | Find small ways to improve your talents | Primary | 3.44 | 1.26 | 17 | **7** | **0.656** | 1.231 | 0.058 | -0.741 |
| Talented_21 | | Teach others about your talent | Primary | 3.44 | 1.16 | 16 | **6** | 0.434 | 1.035 | 0.475 | -0.275 |
| Talented_5 | | Join a group that practices your talent regularly | Primary | 3.40 | 1.25 | 15 | **5** | **0.640** | 1.651 | 0.648 | -0.573 |
| Talented_3 | | Find opportunities to share your talents | Primary | 3.39 | 1.24 | 14 | **4** | **0.646** | 1.353 | 0.176 | -0.665 |
| Talented_8 | | Listen to others when they talk | Filler | 3.38 | 1.14 | 13 | **3** | **0.513** | 1.205 | 0.69 | -0.103 |
| Talented_11 | | Perform your talent whenever you can | Primary | 3.33 | 1.27 | 12 | **2** | **0.661** | 1.661 | 0.452 | -0.686 |
| Talented_14 | | Read books about developing a talent | Primary | 3.21 | 1.26 | 11 | **1** | **0.516** | 1.196 | 0.125 | 0.176 |
| Talented_16 | | Revise something your created in the past | Filler | 3.19 | 1.29 | 10 |  | 0.331 | 0.857 | 0.601 | 0.713 |
| Talented_12 | | Play on a sports team | Filler | 3.17 | 1.21 | 9 |  | 0.411 | 0.898 | 0.09 | 0.009 |
| Talented_10 | | Participate in dance or cheerleading | Filler | 3.16 | 1.32 | 8 |  | 0.196 | 0.557 | 0.241 | 1.100 |
| Talented_1 | | Ask a lot of questions | Primary | 3.13 | 1.08 | 7 |  | **0.512** | 0.994 | 0.952 | -0.126 |
| Talented_6 | | Learn a new language | Filler | 3.08 | 1.26 | 6 |  | 0.388 | 1.123 | 0.415 | 0.400 |
| Talented_9 | | Memorize lists of numbers and facts | Filler | 3.08 | 1.22 | 5 |  | 0.330 | 0.692 | 0.87 | 0.540 |
| Talented_2 | | Borrow equipment to support your talent | Filler | 3.07 | 1.16 | 4 |  | 0.619 | 1.258 | 0.114 | -0.309 |
| Talented_15 | | Remember birthdays and other special days | Filler | 3.07 | 1.29 | 3 |  | 0.132 | 0.557 | 0.465 | 1.179 |
| Talented_7 | | Lift weights | Filler | 3.04 | 1.30 | 2 |  | 0.366 | 0.963 | 0.183 | 0.362 |
| Talented_17 | | Run for a school or class office | Filler | 3.01 | 1.29 | 1 |  | 0.196 | 0.595 | 0.004 | 0.962 |
| *Note*: N = 272. SD = standard deviation; IRT-DS = item response theory discrimination parameter; IRT-DF = Item response theory difficulty parameter; Lambda = standardized factor loading from CFA model positing simple structure; MDS = multidimensional scaling location parameter. Bold numbers indicate top ranked activities. | | | | | | | | | | | |
