# Supplementary material for: Methodological strategies for linking superordinate life goals (values) and daily activities: a cross-sectional online study of adolescents
Source: Front Psychol. 2026 Mar 17;17:1685340. doi: 10.3389/fpsyg.2026.1685340 (PMC13036117; doi:10.3389/fpsyg.2026.1685340)
Supplement: Supplementary file 1 [file Data_Sheet_1.zip › Supplemental Table 1 Helpful Activities.docx]

| **Supplemental Table 1.**  *Activity Rankings by the Four Methods for the Helpful Life Goal* | | | | | | | | | | |
| --- | --- | --- | --- | --- | --- | --- | --- | --- | --- | --- |
|  | | | | | | | | | | |
| **Variable** | **Activity** | **FIT** | **Mean** | **SD** | **Top 21** | **Top 11** | **Lambda** | **IRT-DS** | **IRT-DF** | **MDS** |
| Helpful_6 | Give advice when someone asks for it | Primary | 3.84 | 1.23 | 21 | **11** | **0.644** | 1.869 | -0.542 | -0.674 |
| Helpful_10 | Help with raising money for good causes | Primary | 3.83 | 1.239 | 20 | **10** | **0.720** | 2.331 | -0.455 | -0.688 |
| Helpful_20 | Volunteer to help with special events | Primary | 3.79 | 1.271 | 19 | **9** | **0.723** | 2.621 | -0.451 | -0.639 |
| Helpful_19 | Volunteer at a local charity | Primary | 3.78 | 1.272 | 18 | **8** | **0.726** | 2.751 | -0.383 | -0.653 |
| Helpful_9 | Help someone with their homework | Primary | 3.73 | 1.265 | 17 | **7** | **0.716** | 2.148 | -0.365 | -0.607 |
| Helpful_12 | Mentor younger students | Primary | 3.72 | 1.243 | 16 | **6** | **0.663** | 2.196 | -0.352 | -0.549 |
| Helpful_17 | Share tools and equipment you own | Primary | 3.72 | 1.241 | 15 | **5** | **0.650** | 2.08 | -0.415 | -0.431 |
| Helpful_15 | Run errands for someone | Primary | 3.67 | 1.26 | 14 | **4** | **0.693** | 2.23 | -0.383 | -0.515 |
| Helpful_1 | Be a good listener when someone needs help | Primary | 3.61 | 1.294 | 13 | **3** | **0.693** | 2.196 | -0.165 | -0.609 |
| Helpful_8 | Help neighbors with cleaning their home | Primary | 3.61 | 1.25 | 12 | **2** | **0.624** | 1.421 | -0.176 | -0.452 |
| Helpful_5 | Give a lot of compliments | Filler | 3.57 | 1.225 | 11 | **1** | **0.556** | 1.483 | **-0.252** | -0.185 |
| Helpful_7 | Give small gifts | Filler | 3.49 | 1.129 | 10 |  | 0.533 | 1.354 | -0.070 | -0.029 |
| Helpful_2 | Be cheerful when someone asks for help | Primary | 3.39 | 1.236 | 9 |  | **0.587** | **1.567** | 0.020 | -0.194 |
| Helpful_21 | Watch education programs and documentaries | Filler | 3.33 | 1.224 | 8 |  | 0.221 | 0.752 | 0.244 | 0.569 |
| Helpful_16 | Seek feedback when you perform for others | Filler | 3.27 | 1.206 | 7 |  | 0.453 | 1.424 | 0.253 | 0.231 |
| Helpful_18 | Talk a lot to smart people | Filler | 3.22 | 1.246 | 6 |  | 0.352 | 1.024 | 0.221 | 0.541 |
| Helpful_14 | Read books about developing a talent | Filler | 3.09 | 1.257 | 5 |  | 0.310 | 0.904 | 0.559 | 0.753 |
| Helpful_13 | Perform in skits and improve sketches | Filler | 3 | 1.295 | 4 |  | 0.259 | 0.698 | 0.924 | 0.873 |
| Helpful_3 | Do exercises like jumping jacks | Filler | 2.9 | 1.307 | 3 |  | 0.137 | 0.627 | 1.171 | 1.179 |
| Helpful_4 | Do push-ups, squats, lunges, and planks | Filler | 2.88 | 1.309 | 2 |  | 0.170 | 0.776 | 0.935 | 1.123 |
| Helpful_11 | Invent a game or puzzle | Filler | 2.78 | 1.094 | 1 |  | 0.172 | 0.824 | 1.44 | 0.955 |
| *Note*: N=288. SD = standard deviation; IRT-DS = item response theory discrimination parameter; IRT-DF = item response theory difficulty parameter; Lambda = standardized factor loading from CFA model positing simple structure; MDS = multidimensional scaling location parameter. Bold numbers indicate top ranked activities. | | | | | | | | | | |
