# Supplementary material for: Methodological strategies for linking superordinate life goals (values) and daily activities: a cross-sectional online study of adolescents
Source: Front Psychol. 2026 Mar 17;17:1685340. doi: 10.3389/fpsyg.2026.1685340 (PMC13036117; doi:10.3389/fpsyg.2026.1685340)
Supplement: Supplementary file 1 [file Data_Sheet_1.zip › Supplemental Table 2 Creative Activities.docx]

| **Supplemental Table 2.**  *Activity Rankings by the Four Methods for the Creative Life Goal* | | | | | | | | | | |
| --- | --- | --- | --- | --- | --- | --- | --- | --- | --- | --- |
|  | | | | | | | | | | |
| **Variable** | **Activity** | **FIT** | **Mean** | **SD** | **Top 21** | **Top 11** | **Lambda** | **IRT-DS** | **IRT-DF** | **MDS** |
| Creative_5 | Find small ways to improve your talent | Filler | 3.79 | 1.16 | 21 | **11** | **0.537** | 1.000 | -0.577 | .755 |
| Creative_7 | Join group -practices your talent regularly | Filler | 3.62 | 1.24 | 20 | **10** | **0.586** | 1.658 | -0.271 | .501 |
| Creative_20 | Write down your thoughts and ideas | Filler | 3.55 | 1.21 | 19 | **9** | **0.601** | 1.508 | -0.214 | .468 |
| Creative_4 | Draw sketches of people or things | Primary | 3.53 | 1.23 | 18 | **8** | **0.501** | 1.296 | -0.198 | .877 |
| Creative_15 | Revise something your created in the past | Primary | 3.52 | 1.20 | 17 | **7** | **0.679** | 1.930 | -0.075 | .229 |
| Creative_18 | Take photos of people or things | Primary | 3.52 | 1.17 | 16 | **6** | **0.573** | 1.542 | -0.212 | .289 |
| Creative_21 | Write stories or poems | Primary | 3.51 | 1.27 | 15 | **5** | **0.545** | 1.337 | -0.185 | .651 |
| Creative_10 | Perform in skits and improve sketches | Primary | 3.49 | 1.25 | 14 | **4** | **0.560** | 1.235 | -0.192 | .098 |
| Creative_8 | Learn to play a musical instrument | Primary | 3.47 | 1.28 | 13 | **3** | **0.570** | 1.302 | -0.155 | -.069 |
| Creative_14 | Read books or articles about inventions | Primary | 3.42 | 1.26 | 12 | **2** | **0.640** | 1.624 | -0.058 | -.209 |
| Creative_1 | Ask others about hobbies and interests | Filler | 3.36 | 1.11 | 11 | 1 | 0.415 | 0.798 | 0.331 | .098 |
| Creative_9 | Mentor younger students | Filler | 3.36 | 1.24 | 10 |  | **0.581** | 1.307 | 0.067 | -.091 |
| Creative_13 | Read about creative people | Primary | 3.36 | 1.22 | 9 |  | **0.674** | 1.614 | 0.13 | -.047 |
| Creative_6 | Invent a game or puzzle | Primary | 3.35 | 1.26 | 8 |  | **0.606** | 1.485 | 0.118 | -.369 |
| Creative_16 | Run, jog, ride a bicycle, or swim | Filler | 3.31 | 1.26 | 7 |  | 0.449 | 0.996 | 0.200 | .191 |
| Creative_17 | Stretch or do yoga | Filler | 3.26 | 1.22 | 6 |  | 0.466 | 0.969 | 0.270 | .014 |
| Creative_3 | Compose music | Primary | 3.24 | 1.25 | 5 |  | 0.398 | 0.973 | 0.308 | -.384 |
| Creative_19 | Write computer code | Primary | 3.12 | 1.33 | 4 |  | 0.542 | 1.308 | 0.280 | -1.027 |
| Creative_12 | Play games like chess and strategy games | Filler | 3.11 | 1.27 | 3 |  | **0.624** | 1.576 | 0.395 | -.609 |
| Creative_11 | Play challenging computer games | Filler | 3.09 | 1.17 | 2 |  | 0.466 | 0.964 | 0.605 | -.675 |
| Creative_2 | Attend social events and parties | Filler | 3.04 | 1.17 | 1 |  | 0.398 | 0.991 | 0.771 | -.691 |
| *Note*: N = 383. SD = standard deviation; IRT-DS = item response theory discrimination parameter; IRT-DF = item response theory difficulty parameter; Lambda = standardized factor loading from CFA model positing simple structure; MDS = multidimensional scaling location parameter. Bold numbers indicate top ranked activities. | | | | | | | | | | |
