# Supplementary figures and images for: Methodological strategies for linking superordinate life goals (values) and daily activities: a cross-sectional online study of adolescents
Source: Front Psychol. 2026 Mar 17;17:1685340. doi: 10.3389/fpsyg.2026.1685340 (PMC13036117; doi:10.3389/fpsyg.2026.1685340)

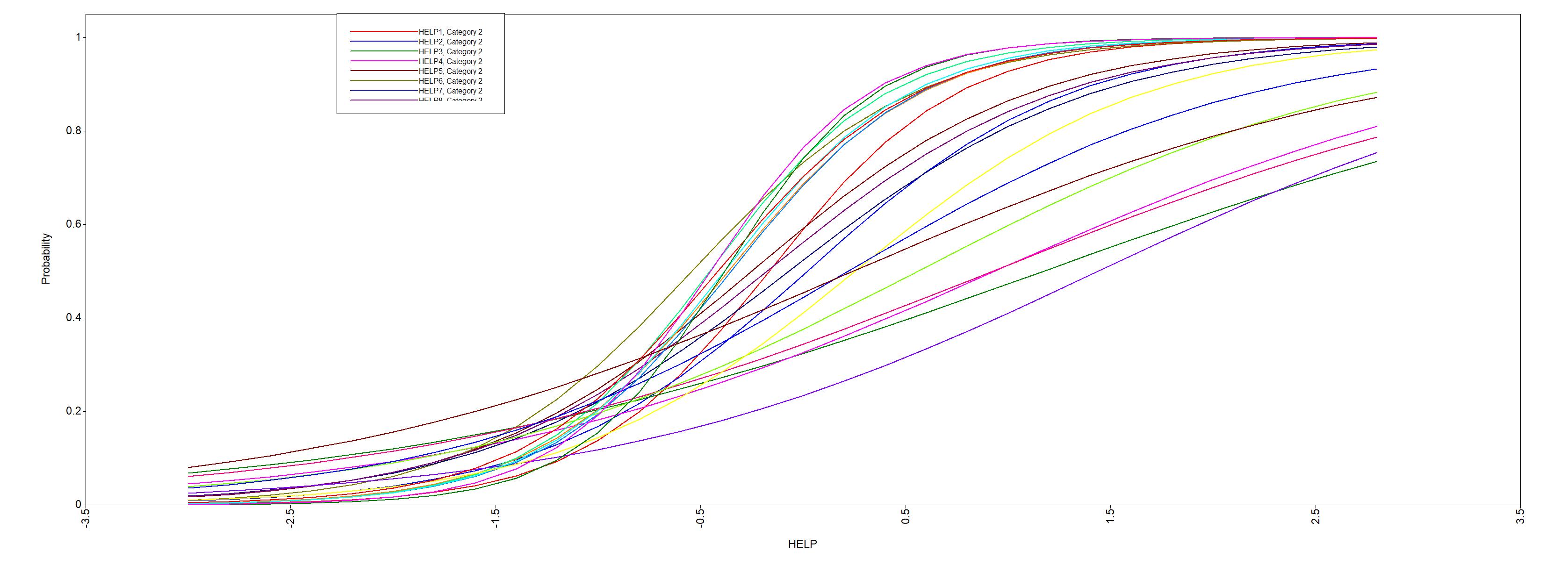

Supplement: Supplementary file 2 [file Image_1.jpg]

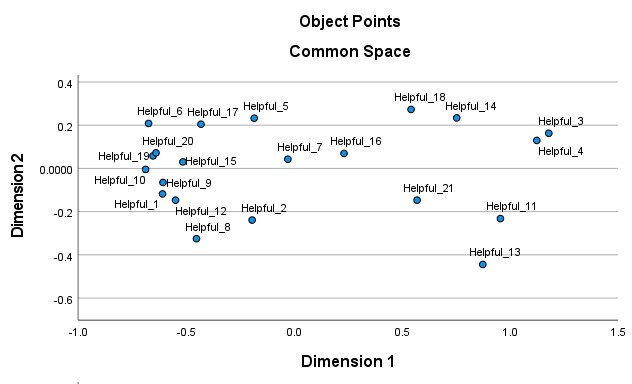

Supplement: Supplementary file 3 [file Image_2.jpeg]
